# EGFR外显子20插入突变型NSCLC治疗的研究进展

**DOI:** 10.3779/j.issn.1009-3419.2023.101.05

**Published:** 2023-02-20

**Authors:** Meiyi XU, Jiawei LUO, Ruilian XU

**Affiliations:** ^1^518020 深圳，暨南大学第二临床医学院（徐美怡）; ^1^The Second Clinical Medical College, Jinan University, Shenzhen 518020, China; ^2^518020 深圳，深圳市人民医院肿瘤科（罗佳伟，许瑞莲）; ^2^Department of Oncology, Shenzhen People’s Hospital, Shenzhen 518020, China

**Keywords:** 肺肿瘤, EGFR外显子20插入突变, EGFR突变, 靶向治疗, Lung neoplasms, EGFR exon 20ins mutation, EGFR mutation, Targeted therapy

## Abstract

肺癌已成为对人类健康威胁最大的肿瘤之一，死亡率位居肿瘤死因之首，非小细胞肺癌（non-small cell lung cancer, NSCLC）占肺癌的80%-85%，晚期NSCLC的治疗以化疗为主，但5年的生存率较低。表皮生长因子受体（epidermal growth factor receptor, EGFR）基因突变是肺癌中最常见的驱动基因变异，而EGFR外显子20插入（EGFR exon 20 insertions, EGFR ex20ins）突变型属于其中一种罕见突变，在所有EGFR突变中占4%-10%，在NSCLC中约占1.8%。近年来，以EGFR酪氨酸激酶抑制剂（EGFR-tyrosine kinase inhibitors, EGFR-TKIs）为代表的靶向治疗已经成为晚期NSCLC患者一个重要的治疗选择，然而EGFR ex20ins突变型的NSCLC患者对大部分EGFR-TKIs治疗并不敏感。目前，部分针对EGFR ex20ins突变的靶向药物取得了明显疗效，部分药物仍在临床研究中。本文将针对EGFR ex20ins突变的各种治疗方法及其疗效进行阐述。

## Abstract (en)

Lung cancer has become one of the most dangerous cancers to human health and the mortality rate is the highest among all the causes of cancer death. Non-small cell lung cancer (NSCLC) accounts for about 80%-85% of lung cancer. Chemotherapy is the main treatment for advanced NSCLC, but the 5-year survival rate is low. Epidermal growth factor receptor (EGFR) mutations are the most common driver mutations in lung cancer, but EGFR exon 20 insertions (EGFR ex20ins) mutation belongs to one of the rare mutations, accounting for about 4%-10% of overall EGFR mutations, thus around 1.8% of advanced NSCLC patients. In recent years, targeted therapies represented by EGFR tyrosine kinase inhibitors (TKIs) have become an important treatment option for patients with advanced NSCLC, however, NSCLC patients with EGFR ex20ins mutation are not sensitive to most of EGFR-TKIs treatments. Currently, some of the targeted drugs for EGFR ex20ins mutation have achieved significant efficacy, while some of them are still under clinical investigation. In this article, we will describe various treatment methods for EGFR ex20ins mutation and their efficacy.

根据2020年全球癌症统计数据^[1]^显示，肺癌全球发病率居第2位，每年新发病例达220万，占新发肿瘤总数的11.4%，而肺癌死亡率位居肿瘤死因之首，占肿瘤死亡总数的14.3%。肺癌已成为对人类健康威胁最大的肿瘤之一，其中非小细胞肺癌（non-small cell lung cancer, NSCLC）是肺癌的主要病理类型，占80%-85%，且大部分患者在发现时已处于疾病晚期。以铂类为基础的化疗是晚期NSCLC的主要治疗手段，但其毒性及不良反应限制了临床应用^[2]^。近年来随着基因检测技术的进步，表皮生长因子受体（epidermal growth factor receptor, EGFR）逐渐被认识，发现我国38.1%的NSCLC患者存在EGFR突变，且有研究^[3,4]^表明亚洲肺腺癌患者中EGFR突变率高达47.9%。多项研究^[5,6]^表明以EGFR酪氨酸激酶抑制剂（EGFR-tyrosine kinase inhibitors, EGFR-TKIs）为代表的靶向治疗不但显著延长了晚期NSCLC患者的无进展生存期（progression-free survival, PFS），同时也改善了患者的生活质量，这使得晚期NSCLC患者的治疗发生了革命性的变化。

但由于EGFR外显子20插入（EGFR exon 20 insertions, EGFR ex20ins）结构多变加上检测手段的局限，使其检出率低、诊断率低、治疗方法缺乏。现有的EGFR-TKIs、化疗和免疫治疗对这种突变的患者治疗有限，预后较差。目前，有大量针对EGFR ex20ins突变的靶向药物临床研究正在进行，部分已经获批于临床使用，部分虽然仍在研究阶段，但已取得了显著的成果。本综述将结合近几年针对EGFR ex20ins突变型NSCLC诊疗现状的研究进展进行总结和回顾。

## 1 EGFR ex20ins突变分子特征及流行病学

EGFR基因由28个外显子组成，其中大多数突变发生在外显子18-21。常见突变包括外显子19框内缺失（exon 19 inframe deletion, ex19del）和外显子21 L858R改变（exon 21 L858R alterations, ex21L858R），占所有EGFR突变的80%-85%，这两类经典的EGFR突变对一代EGFR-TKIs（厄洛替尼、吉非替尼）、二代EGFR-TKIs（阿法替尼、达可替尼）及三代EGFR-TKIs（奥西替尼）药物具有很好的敏感性，其余EGFR突变称为罕见突变。在罕见突变中，以EGFR ex20ins突变最为常见，在NSCLC中约占1.8%，在所有EGFR突变中占4%-10%，是仅次于ex19del及ex21L858R的第三常见突变^[7]^。EGFR外显子20由762位-823位氨基酸组成，包含两个重要区域：762位-766位氨基酸的调节性C螺旋（C-helix）以及由紧邻着的767位-774位氨基酸形成的磷酸结合环P-loop。EGFR ex20ins主要发生于C-helix之后的Met766-Cys775（约90%）^[8]^。目前，已鉴定了超过60种EGFR ex20ins激活突变，大多数是通过紧邻C-helix的loop环插入1个-4个氨基酸，使得C-helix进入激活状态，然后将C-helix及磷酸结合环P-loop推入药物结合口袋中，诱导受体活性构象，从而以不依赖配体的方式激活受体^[9]^。而这一立体的位阻效应阻碍了EGFR与TKIs的结合。所以在C螺旋之后的氨基酸插入对EGFR-TKIs药物耐药，而少见的发生在C-helix之前（Glu762-Tyr764）的插入可对EGFR-TKIs药物有一定的敏感性。有研究^[10,11]^在比较EGFR ex20ins D770insNPG和EGFR T790M以及野生型EGFR的3D模型发现，EGFR ex20ins与EGFR T790M结构类似，这解释了对一代非共价EGFR-TKIs耐药及对一、二代EGFR-TKIs不敏感的原因。第三代EGFR-TKIs对母环创新，不饱和丙烯酰基与C79形成共价(不可逆结合)，去掉了头部的芳香基团，解决了EGFR ex20ins及T790M导致的空间位阻问题。

## 2 检测方法

临床上常用的EGFR ex20ins检测方法主要包括聚合酶链反应（polymerase chain reaction, PCR）法、Sanger测序法和下一代测序（next generation sequencing, NGS）等。PCR法是通过检测特定片段是否存在从而推断基因是否发生突变，是传统的检测基因突变的方法，但只能检测已知的突变类型，EGFR ex20ins突变结构多变，PCR能覆盖的类型少，不是全面检测EGFR ex20ins突变的最佳方法^[12,13]^。Sanger测序法是针对已知致病基因的突变位点设计引物，进行PCR扩增直接测序，可直接检测已知和未知的突变，但检测灵敏度较低。NGS是基于PCR和基因芯片发展而来的DNA测序技术，二代测序在DNA复制过程中通过捕捉新添加的碱基所携带的特殊标记（一般为荧光分子标记）来确定DNA序列，也能直接检测已知和未知的突变，且灵敏度高，可以实现大规模、高通量测序的目的，但费用高昂，需要含有足够质量和数量的核酸标本。可见每种检测方法都有其优缺点，在临床上应根据实际情况来选择检测方法。

## 3 EGFR ex20ins突变型NSCLC的治疗

### 3.1 一线/二线化疗

目前含铂双药化疗仍然是EGFR ex20ins突变型NSCLC患者的标准治疗手段。有研究^[14]^称由于携带EGFR ex20ins突变型NSCLC患者对一、二代TKIs不敏感，甚至接受化学治疗比一、二代TKIs获益得更多，客观缓解率（objective response rate, ORR）为58%-63%，PFS为6.3个月。另外有数据^[15]^表明这部分患者一线接受含铂双药化疗的中位PFS为6.4个月，而接受一代TKIs治疗的中位PFS仅为2个月，且在二线治疗中接受化疗对比使用EGFR-TKIs治疗的患者中位PFS为4个月 vs 2个月。另一项纳入我国119例EGFR ex20ins突变型NSCLC患者的数据^[16]^显示，培美曲塞或许应当作为化疗的优先选择，因为含有培美曲塞方案和非培美曲塞方案的治疗的中位PFS分别为5.5个月和3.0个月；含有培美曲塞方案的总生存期（overall survival, OS）同样更长（25.0个月 vs 19.6个月）。但化疗药物的副作用使患者难以耐受，这也限制了化疗在临床上的使用。

### 3.2 靶向治疗

#### 3.2.1 EGFR-TKIs类药物

##### 3.2.1.1 一代TKIs类药物（吉非替尼、厄洛替尼）

这是一类可逆的ATP竞争性抑制剂，可以选择性抑制ex19del及ex21L858R突变型EGFR，但是对ex20ins突变却几乎没有疗效。既往有研究^[17]^显示，晚期NSCLC并携带EGFR ex20ins突变的患者接受一代EGFR-TKIs治疗的中位PFS仅为1.9个月，与安慰剂相差无几。但并非所有插入突变的患者对一代EGFR-TKIs不敏感，少部分突变类型A763 _Y764insFQEA突变就显示出对一代EGFR-TKIs很高的结合亲和力^[18,19]^。

##### 3.2.1.2 二代TKIs类药物（阿法替尼、达可替尼）

这是一类不可逆的EGFR抑制剂，且对EGFR受体的阻断更为彻底。但根据研究结果显示疗效并不佳。来自Journal of Thoracic Oncology的一项纳入70例携带ex20ins突变并接受阿法替尼治疗的研究^[20]^显示其ORR为24.3%，有轻微的治疗活性。尽管体外研究数据^[21]^发现阿法替尼联合西妥昔单抗对EGFR进行双重阻断可以提高疗效，但缺乏临床数据的支持。实际上，在经典突变领域中，两项前瞻性研究^[22,23]^已经证实该联合治疗策略并不可行。

##### 3.2.1.3 三代TKIs类药物（奧西替尼、伏美替尼）

这是一类不可逆的、选择性作用于EGFR敏感突变和T790M突变的EGFR抑制剂。临床前研究^[24]^报道，奥西替尼对EGFR ex20ins突变有效。在一项评价奥西替尼单药治疗的疗效和安全性的研究^[25]^中，共纳入6例EGFR ex20ins突变患者，患者每天服用1次80 mg剂量的奥西替尼。4例患者达到部分缓解（partial response, PR），其余患者为疾病稳定（stable disease, SD），中位PFS为6.2个月。为评估奥西替尼对一线化疗失败后EGFR ex20ins突变的NSCLC患者的疗效，韩国的一项多中心、II期临床研究（LU17-19）^[26]^纳入了15例患者：3例患者为二线治疗，12例患者为≥3线治疗。结果显示中位PFS为3.5个月，6个月时的疾病控制率（disease control rate, DCR）为 31.1%。有研究^[27]^对EGFR ex20ins突变的NSCLC患者进行了奥西替尼剂量增加到160 mg的单臂II期研究。结果发现，20例患者的ORR为25%，中位PFS为9.7个月，中位持续缓解时间（duration of overall response, DOR）为5.7个月。这一结果表明，高剂量奥西替尼可作为耐受性良好的患者的治疗选择。但是此研究数据存在不合理的地方：中位DOR远远小于中位PFS，考虑到小样本研究生存曲线往往呈现为阶梯状，表明中位PFS的估算可能并不稳定。实际上，在Lung Cancer杂志发表的一项研究^[28]^发现，奥西替尼加量治疗这部分患者的ORR和DCR分别只有6.5%和53.2%，中位PFS仅有2.3个月，并未显示任何治疗效果，与前述研究结论截然相反，这两项研究表明要加大样本量进一步研究才能确切了解高剂量的奥西替尼是否对EGFR ex20ins突变型NSCLC患者真正有疗效。而伏美替尼是我国原研的第三代EGFR-TKIs药物，在针对EGFR ex20ins突变患者的FAVOUR Ib期研究中，初步数据^[29]^显示伏美替尼240 mg一线治疗EGFR ex20ins突变型NSCLC患者的ORR达70%，DCR均为100%。另一个针对≥2线的EGFR ex20ins突变型NSCLC患者的研究^[30]^显示，15例接受伏美替尼治疗的患者总体ORR为53.5%，DCR为100%，3个月PFS率为100%。且以上2项研究均显示伏美替尼的安全性、耐受性良好，未发现≥3级不良事件。但以上2项研究的纳入样本量较小，PFS和OS数据仍需进一步跟进和更大样本量验证，期待伏美替尼能带来更好的消息。

#### 3.2.2 新型靶向治疗

##### 3.2.2.1 Poziotinib（波奇替尼）

Poziotinib是EGFR/人表皮生长因子受体2（human epidermal growth factor receptor 2, HER2）/HER4的不可逆抑制剂，化学结构上与第一个EGFR/HER2双抑制剂拉帕替尼为同一母核。Cha及其同事^[31]^发现，波奇替尼与EGFR和HER2共价结合，可以抑制携带有EGFR经典突变、T790M突变以及HER2高表达的NSCLC细胞株。Elamin等^[32]^研究发现，在Ba/F3模型中，波奇替尼对EGFR ex20ins突变具有体外活性。相比于阿法替尼、奥西替尼等，波奇替尼的半数抑制浓度（half-maximal inhibitory concentration, IC_50_）值最低，增殖抑制作用比奥西替尼强100倍，比阿法替尼强40倍。ZENITH20是一项多中心、多队列的II期临床试验^[33]^，队列1纳入的是经治的EGFR ex20ins突变NSCLC患者，接受波奇替尼16 mg qd口服治疗。研究结果显示，ORR为14.8%，DCR为68.7%，中位PFS为4.2个月，中位DOR为7.4个月。但88%的患者因不良事件暂停用药，68%的患者因此减量，10%的患者永久停药。考虑到波奇替尼原给药方案的严重不良反应比例较高，且其半衰期仅为7.9 h，ZENITH20-5研究^[34]^中队列采用了16 mg qd或8 mg bid的对比给药方案，结果显示8 mg bid组（n=19）的ORR为31.6%，DCR为78.4%，明显高于16 mg qd组（n=19）的15.8%和52.6%，而严重不良反应比例也明显降低（23% vs 35%）。MD Anderson癌症中心也针对波奇替尼开展了一项单中心II期临床研究^[35]^，入组了既往接受过多线治疗，且携带EGFR或HER2的外显子20插入的NSCLC患者，结果显示总体ORR达到32%，中位PFS为5.5个月，不良事件发生比例较高且严重。2022年年底，波奇替尼的上市申请遭到美国食品药品监督管理局（Food and Drug Administration, FDA）的拒绝。波奇替尼的问题可能在于研究数据的ORR低、DOR短，且推荐剂量的安全性较差，波奇替尼被FDA拒绝上市后也影响了其后续进一步的研发。

##### 3.2.2.2 Mobocertinib（TAK-788或AP32788）

Mobocertinib是一种新型、高选择性靶向于EGFR的不可逆TKIs。来自一项I期/II期多中心研究（NCT02716116）的初始临床数据^[36]^显示，在既往接受过含铂化疗的EGFR ex20ins的晚期NSCLC患者中，接受Mobocertinib（160 mg/d）治疗的ORR为43%，中位DOR为13.9个月，DCR为86%，中位PFS为7.3个月。基线有脑转移和无脑转移的患者，ORR分别为56%和25%，中位PFS分别为10.2个月和3.7个月，安全性良好。另一项在2022年欧洲肿瘤内科学会（European Society for Medical Oncology, ESMO）大会上公布的I期/II期研究^[37]^结果显示其经确认的ORR为28.1%，DCR为78%，中位PFS为7.3个月，中位OS为20.2个月。2023年1月11日，Mobocertinib在我国获批上市用于治疗含铂化疗期间或之后进展且携带EGFR ex20ins突变的局部晚期或转移性NSCLC患者，成为国内首个用于EGFR ex20ins突变的药物。正在进行的III期EXCLAIM-2研究^[38]^对Mobocertinib与铂类双药化疗在初治患者中的疗效进行了评估，这是第一项评估EGFR ex20ins靶向治疗与一线标准化疗疗效差异的前瞻性研究。

##### 3.2.2.3 Amivantamab（JNJ-372）

Amivantamab是另一种全人源化EGFR-MET双特异性抗体，作用机制与既往的口服EGFR-TKIs截然不同。该抗体采用的是非对称形式，即其中一个Fab结合MET靶点，另一个Fab结合EGFR靶点，可绕过TKIs结合位点的抗性，同时与不同受体的胞外段区域结合，促进受体-抗体复合物的降解，并触发抗体依赖的细胞介导的细胞毒性作用（antibody-dependent cell-mediated cytotoxicity, ADCC）从而发挥对肿瘤的杀伤作用^[39]^。在CHRYSALIS I期临床研究^[40]^中，共计258例患者接受推荐剂量治疗，即1,050 mg（体重<80 kg）或1,400 mg（体重≥80 kg），其中有81例EGFR ex20ins型NSCLC患者可以进行疗效评估，既往均接受过铂类药物治疗，25%的患者接受过EGFR-TKIs的治疗，22%的患者既往有脑转移病史，最终结果显示ORR为40%，中位DOR为11.1个月，中位PFS为8.3个月，中位OS为22.8个月，与治疗相关的3级不良事件共18例（16%）。Amivantamab于2021年5月被FDA批准用于含铂药物治疗后或者正在治疗中的EGFR ex20ins NSCLC患者，成为少有的只依据I期临床数据便能获批的药物^[41]^。总体来说，Amivantamab通过新的双靶点作用机制，具有良好的疗效和持久的缓解，且其安全性在可接受的范围内，活性比EGFR ex20ins既往的治疗方案更好。

##### 3.2.2.4 CLN-081

CLN-081是一种不可逆的、新型口服EGFR-TKIs，具有特殊的吡咯并嘧啶结构，其特异性支架可安装在EGFR铰链区ATP结合位点上，CLN-081对EGFR ex20ins具有选择特异性亲和力，其IC_50_比（野生型/突变型）>5倍以上。2022年美国临床肿瘤学会（American Society of Clinical Oncology, ASCO）上报告了CLN-081治疗EGFR ex20ins突变型NSCLC的多中心I期/IIa期试验^[42]^数据，CLN-081-001是一项剂量递增和扩展试验，其研究对象是先前接受过含铂药物的基础化疗复发或转移性EGFR ex20ins突变型NSCLC患者。在剂量递增队列中，在剂量为≤65 mg、100 mg、150 mg的组别中，中位DOR分别为>19个月 vs >21个月 vs 7个月，中位PFS对比为8个月 vs 12个月 vs 8个月。安全事件方面：15%的患者出现治疗相关的不良反应，最常见的是皮疹（74%）和腹泻（27%）。研究前已基线存在中枢神经系统病变的3例患者中，2例患者获得PR（其中1例患者治疗时间已超过1年），另1例患者出现疾病进展。综上所述，CLN-081在100 mg bid剂量显示出令人欣喜的临床有效率和安全性，FDA已指定CLN-081用于EGFR ex20ins NSCLC患者的治疗。目前正进行IIa期临床试验，期待后续临床数据公布。

##### 3.2.2.5 Sunvozertinib（DZD9008）

DZD9008是一种口服的、高效、不可逆的选择性EGFR-TKIs，是靶向作用于EGFR/HER2 ex20ins的小分子化合物。对已知EGFR突变的类型均有效且对野生型EGFR抑制作用较弱。在2021年ASCO年会，迪哲生物公布了DZD9008 I期临床研究安全性评估队列（102例，EGFR突变或HER2突变）和疗效评估队列（56例，EGFR 20ins）的研究数据，超过90%的入组患者接受过化疗，超过40%的患者有脑转移，超过40%的患者接受过EGFR-TKIs治疗，另外有30%患者接受过免疫治疗。疗效评估队列中有31例患者接受300 mg qd的治疗，ORR为41.9%，DCR为90.3%，3级以上治疗相关不良反应发生率为33.3%。在接受200 mg qd的11例患者中，ORR为45.5%，DCR为81.8%，3级以上治疗相关不良反应发生率为6.3%。从数据上看，降低100 mg剂量对DZD9008疗效似乎影响不大且耐受性明显提高。23例入组时存在稳定脑转移病灶的患者有7例PR，至数据截止时间，该研究的中位DOR>3.5个月，在23例PR的患者中，仍有15例患者处于SD^[43]^。从以上的研究数据来看，DZD9008在EGFR ex20ins突变型NSCLC患者中显示出良好的安全性和疗效。基于以上数据，FDA于2022年年初授予DZD9008突破性疗法认定。2022年ASCO会上，两项进行中的I期/II期研究（WK-KONG1和WUKONG2）汇报了研究^[44]^结果，入组标准是既往接受过含铂化疗±程序性死亡配体1（programmed death ligand 1, PD-L1）治疗后疾病进展的EGFR ex20ins突变患者，接受DZD9008 50 mg-400 mg qd的治疗。在100 mg、200 mg、300 mg和400 mg队列中，确认的ORR分别为50.0%、55.6%、44.8%和22.2%，6个月PFS率分别为50.0%、53.3%、44.6%和44.4%。2022年ESMO大会公布了中国注册、多中心II期WU-KONG6研究^[45]^的初步结果，300 mg剂量条件下经确认的ORR为59.8%，基线有脑转移的患者经确认的ORR为48.4%。无论既往是否接受过PD-L1的治疗，DZD9008的疗效和安全性相当。期待后续大样本量研究令人欣喜的结果。

### 3.3 免疫治疗

真实世界里，EGFR ex20ins突变型NSCLC一线治疗多数采用含铂化疗方案，免疫治疗在二线和三线使用更为多见。2021年ASCO年会，一项研究^[46]^汇报了真实世界中携带EGFR ex20ins突变型NSCLC的疗效及安全性的数据。研究显示分别有11例一线接受免疫单药及16例一线接受免疫联合化疗的患者，其ORR为9.1% vs 18.8%，中位PFS为3.1个月 vs 4.5个月，中位OS为11.0个月 vs 11.3个月；而32例二线接受免疫单药治疗的患者，ORR仅为3.1%，中位PFS为3.3个月，中位OS为8.1个月。但刊登在Clinical Lung Cancer杂志的另一项研究^[47]^纳入携带EGFR ex20ins突变型晚期NSCLC患者有6例，接受免疫单药治疗后的ORR分别为50%，中位PFS为4.8个月，结论与前者完全不同。Metro等^[48]^的研究结果显示，接受免疫治疗的EGFR ex20ins突变型晚期NSCLC患者中位PFS为2个月，中位OS为5.3个月，免疫治疗的效果欠佳。以上几项研究纳入样本量较小，得出的结论也不尽相同，期待后续有更大样本量的研究结果能给出确切的结论。

## 4 总结与展望

EGFR ex20ins突变在肺癌中虽占比不大，但是由于肺癌患者的基数大，这类患者仍然是不可忽视的庞大群体。积极探寻更佳的治疗策略，无疑可以为EGFR ex20ins突变型NSCLC患者带来更多的生存获益。目前化疗仍是标准一线治疗，以培美曲塞为基础的化疗稍占优势。一代及二代TKIs均未见疗效，三代TKIs奥西替尼通过药物加量或可展示一定的疗效，需进一步的研究去验证，而国产原研三代TKIs伏美替尼的研究结果显示疗效值得期待。免疫治疗在这部分患者的研究很少，现有的研究数据提示疗效欠佳，但研究纳入的样本量不够，尚未能根据现有的研究数据得出确切的结论。近年来，随着新一代测序技术和分子检测技术的更新和发展，人们对EGFR罕见突变有了更进一步的认识。越来越多的临床试验专注于研究此类突变，因此出现了多种罕见驱动基因的新型靶向药物。Amivantamab基于真实世界的实验研究数据已经证明，对于既往曾接受过含铂类药物化疗后进展的EGFR ex20ins突变型晚期NSCLC患者表现出良好的临床活性及安全性，期待数据的进一步更新。TAK-788凭借亮眼的研究结果及良好的安全性已于我国上市。CLN-081和DZD900都因其令人惊喜的I期、II期临床数据被FDA授予突破性疗法，更多研究仍然在进行中，希望后续大样本量的临床研究数据仍能获得满意的结果。波奇替尼也有一定的抗肿瘤活性，但因其安全性欠佳，新药上市申请已被FDA拒绝。由于EGFR结构的多样性和复杂性，EGFR ex20ins漏检率偏高。虽然NGS的进一步发展提供了更全面的EGFR ex20ins突变检测，是目前检测的金标准，但由于成本大及对标本要求高，未能在临床上进一步推广。另一方面，绝大部分目前批准的药物适应证以及临床研究都是在二线及以上的后线患者中进行，对很多患者的一线治疗需求无法满足。此外，因耐药发生率高，深入了解耐药机制，克服耐药及减少药物的毒副反应也值得关注。针对上述问题，还需要进一步深入的研究，期待更多的治疗策略使EGFR ex20ins突变的患者接受到更为个体化、精准化的治疗。
